# Reducing Job-Related Distress to “Burnout” Raises Concerns

**DOI:** 10.1177/21650799261448183

**Published:** 2026-05-04

**Authors:** Renzo Bianchi

**Affiliations:** 1 Department of Psychology, Norwegian University of Science and Technology (NTNU)

**Keywords:** burnout, anxiety, depression, job stress, symptomatology

When stress at work becomes insurmountable, it can give rise to a range of symptoms commonly referred to as job-related distress (Bianchi et al., 2024; Niedhammer et al., 2020). Such symptoms include dysphoria, loss of interest and motivation, fatigue, worry, social withdrawal, feelings of failure and worthlessness, cognitive impairment, psychomotor and neurovegetative alterations, and, in severe cases, suicidality (Ringwald et al., 2025; Sen, 2022; Willner et al., 2013).

Despite the well-documented occurrence of these symptoms among workers facing unmanageable stress, research on the distress of health professionals has relied heavily on the construct of “burnout,” which captures only a subset of this symptomatology through its focus on *exhaustion*, *distancing*, and *inefficacy* (Maslach et al., 2016; World Health Organization, 2019). This focus tends to relegate other manifestations of distress to the status of secondary correlates, leaving them unaddressed even when central to workers’ suffering and functioning. This is particularly disquieting when dealing with symptoms such as suicidality (Sen, 2022). An elevated risk of suicide has long been identified among health professionals (Olfson et al., 2023; Zimmermann et al., 2024).

Importantly, the burnout construct’s focus on exhaustion, distancing, and inefficacy was not derived from a systematic examination of workers’ symptomatology, nor was it guided by theory (Bianchi & Sowden, 2024). Rather, this focus emerged from preconceptions and arbitrary choices, subsequently reified by burnout scales (Bianchi & Schonfeld, 2025; Rotenstein et al., 2018). The burnout construct crystallized around exhaustion, distancing, and inefficacy, but it could just as well have centered on other distress symptoms, like dejected mood and feelings of helplessness (Bianchi & Schonfeld, 2025; Sen, 2022).

Given the limitations of the burnout framework, occupational health specialists (OHS) are increasingly turning to more comprehensive indicators of job-related distress, such as (work-anchored) anxiety and depression. Unlike burnout, these indicators are diagnostically framed, thus offering clearer criteria for decision-making and action. Assessments of depressive symptoms routinely include screening for suicidal ideation. Crucially, an anxiety-depression framework enables investigators and OHS to establish normative benchmarks and identify workplaces with abnormally high rates of clinically significant distress, thereby supporting both individual care and organizational accountability.
